# Event and Comment

**Published:** 1907-05-15

**Authors:** Paul Cassidy

**Affiliations:** Covington, Ky.


THE
### DENTAL REGISTER.

Vol. LXI. May 15, 1907. No. 5.

### EVENT AND COMMENT.

### Dental Anesthetics.

The communication which follows we give prominent space in this issue as it presents another view of the discussion of this subject which we presented at considerable length in our March issue. It is a subject of vital interest to every dentist, and one that we ought not to have two opinions about, and yet like many other matter of technical importance it is a subject that is very likely to be individualized. The idea that every one can get the best results from the anesthetic he most uses, shows that there is a value to be attached always to the technical aspect of the medical science part of our work that we do not take into account. We may understand the theory of anesthesia, but unless we practice it continuously we shall never feel safe in the use of even the most harmless drugs. In this connection it may be well to report the death of a lady in Grand Rapids, Mich., February 22nd, 1907. The patient took somnoform to have six teeth extracted, which was successfully accomplished. The patient, however, only partially recovered and died in the operating chair. It is too bad that we can never get the details of these sudden deaths from anesthetics, as the actual cause of the death may be attributed too often to the wrong cause.

EDITOR DENTAL REGISTER.

I shall be glad if you will allow me a little space in your valuable journal to make a few comments on a recent article on anesthetics that appeared in your March Issue and to correct what it seems to me some erroneous statements and conclusions.

Dr. Straith's paper, is replete with valuable points and suggestions and he is certainly to be highly commended for his energy and the time and care he must have spent in its preparation.

Anesthesia it seems to me has been woefully neglected by the teachers in our Colleges and by Dentists themselves. This, the one procedure in dental practice in which is most seriously and often involved the risk of human lives and is the one thing that is most frequently treated with indifference by the Colleges, students, dentists and the State Boards of Examiners. The latter will ask the prospective practitioner inappropriate and useless questions without number regarding the blood and nerve supply of the big toe; they will demand a thorough knowledge of reflexes, of the articulation, insertion and so on "ad nauseam" of muscles and bones throughout the body, but seldom a word regarding the applicant's knowledge of anesthetics and their actions. The State Boards require the insertion of gold and amalagam fillings, of porcelain and gold inlays, of crowns and bridges, but never think of demanding the applicant to administer the general anesthetics that they are about to give him the authority of administering ad libitum.

Dr. Straith has done well, and you have done well in editing and publishing this most interesting article on a subject that we all have so little knowledge of and about which, we all feel so very learned.

Dr. Straith's remarks regarding the absurdity of a patient suggesting any point whatsoever to a physician in the treatment of a case, are very unfortunate it seems to me. I was associated for over three years with a very competent physician and found that he was required to swallow his pride quite as frequently as I was mine.

It seems to me that there are only two points to be considered in the selection of an anesthetic, instead of the three that are mentioned in the paper. Safety and efficiency are all that I should give a thought to. The local method of anesthetic is an absolute failure in the vast majority of cases for the extraction of teeth. And there is a great reason for this fact. Fully ninety percent of the teeth that demand extraction are surrounded by tissues in a state of some degree of inflammation, in which cases local anesthesia is more or less contra-indicated, for it is an indisputable fact that in just the degree that tissues in any part of the body are inflamed, in just that same degree will local anesthesia in those tissues be a failure. The contention, then, of some of the gentlemen who replied to Dr. Straith's letter of inquiry that local anesthesia can displace the general method quite satisfactorily can not be demonstrated.

There is another point to be considered in the comparison of local and general anesthetics, and that point is safety. Statistics will prove to those who take the trouble to investigate that local anesthesia has a very high death rate; that many mute voices are testifying to the incompetency and criminal ignorance of those who have used and are continuing to use cocaine, stovaine and other local agents.

In his reference to the early history of general anesthetics, Dr. Straith makes an error of mentioning "liquid gas." Nitrous Oxid has no especial claim to the title of gas. All general anesthetic agents have the same claim that it has. Somnoform, Eythl Clorid, Ether and Cloroform are gases condensed and remain liquids only under restraint. By continually referring to nitrous oxid as gas, we as dentists are merely fostering in the public mind that ignorance which we are always pretending to enlighten.

Whether Somnoform is as safe as Nitrous Oxid or not, we cannot now infallibly say. Certain it is that in over Six Hundred Thousand administrations in this country there have been reported only three deaths. It has since been learned that in one of these three Ethyl Chlorid, and not Somnoform, was used. In both of the other cases death occured after a re-administration. This is rather a clear record for an anesthetic agent which the article and its discussion lead us to believe is highly dangerous.

A man accustomed to Nitrous Oxid is likely to be alarmed at the first administration of Somnoform that he witnesses. This, however, is due entirely to his lack of knowledge and not from any danger in the anesthetic itself. The man accustomed Nitrous Oxid is alarmed at the rapidy with which anesthesia is induced; he is alarmed at the profound state of that anesthesia—quite different from a high percentage of the cases under Nitrous Oxid; he is also alarmed at the duration which is very much longer that under Nitrous Oxid. If he crawls within his shell and refuses to investigate, with conscious pride he condemns a valuable agent whose only fault is his own lack of knowledge.

Again, in the discussion of the paper, Dr. Loucks tells us that Nitrous Oxid is far safer than Somnoform because of the simple fact that cyanosis appears early under it and we can then cease the administration and run into no dangers whatsoever. For the information of the Doctor and any of his misinformed readers I would say that cyanosis has no bearing whatsoever on the case. The man who ceases the administration of Nitrous Oxid when cyanosis appears had better give up practice, close his shop and move out of town, for he is not only buncoeing his patients but he is also buncoeing himself.

Cyanosis is most emphatically not a sign of anesthesia under Nitrous Oxid. In many cases it is never manifest and the man who waits for its appearance would have his patient ready for the undertaker before ceasing administration. On the other hand, it usually is manifest and in the initial stages of introduction, at that. If, at its appearance, we begin to operate, pain is acute, there is partial consciousness and the patient has never been anesthetized at all. The fear of danger onthe part of the operator at the first sign of cyanosis is the principle cause of the many failures under Nitrous Oxid.

I have at my command continually both Somnotorm and Nitrous Oxid. For three years I have given preference to Somnoform because of self-evident facts. Notwithstanding unsupported evidence to the contrary it has proven itself a safe agent. I have fewer alarming cases under it than under Nitrous Oxid; I obtain a longer and more profound period of anesthesia; attending friends are not so greatly alarmed and I have fewer complaints of after sickness. When I know that on account of the weakened or delicate condition of the patient any operation would be attended with risk, I advise its performance at the home, and, as the Somnoform outfit is portable, I administer this agent in all such cases.

The fact that Somnoform is a commercial article should not by any means place it under the ban. Do the gentlemen who condemn it for this reason realize that Park, Davis & Co's. Adrenalin Chlorid is also a commercial article and do they, to be consistent, decry its use. If they do not use Parke, Davis & Co.'s product they most certainly use some other commercial article of the same nature, which is probably not so pure or so good.

Does it stand to reason that the promoters of Somnoform, after making a place for it in the medical and dental professions, would ever contemplate the changing of the formula as has been suggested they might do? Does any one who is normal and sane ever deliberately knock down the props which support him and which he has taken great pains and much time to erect.

Has it ever occured to any of you that anyone of the several manufacturers of Nitrous Oxid for the trade could combine with it deleterious substances that would diminish cyanosis and give a more profound and tranquil anesthesia? I do not say that any of them would do it, but then neither has anyone the moral right to say that the producers of

Somnoform would do that same thing. A manufacturer of Nitrous Oxid could combine it with other things and announce to the profession that by virtue of the unusual purity of his product there would be a tar more satisfactory anesthesia under it than under that of any other manutacturer. On demonstration he could prove his claims and the unwary practitioner might use that same product tor ever so long a time, and, confident ot its supposed satety, continue its use in his ignorance and egotism. How do we know that, in the administration ot Nitrous Oxid now, we are not using some such product? Has anyone the capacity, time or inclination to test and analyze the contents ot each cylinder as it comes to him? I would most emphatically say not.

I sincerely hope that Dr. Straith's paper will arouse a lengthy discussion and will be but the begining of an agitation that will result in a more thorough and complete knowledge ot this most vital subject,

Respecttully,

Paul Cassidy, A.B., D.D.S.

Covington, Ky., April 11, 1907.

